# Ectopically expressed glutaredoxin ROXY19 negatively regulates the detoxification pathway in *Arabidopsis thaliana*

**DOI:** 10.1186/s12870-016-0886-1

**Published:** 2016-09-13

**Authors:** Li-Jun Huang, Ning Li, Corinna Thurow, Markus Wirtz, Rüdiger Hell, Christiane Gatz

**Affiliations:** 1 Albrecht-von-Haller-Institute for Plant Sciences, Molecular Biology and Physiology, Georg-August-University Göttingen, Julia-Lermontowa-Weg 3, 37077 Göttingen, Germany; 2 Centre for Organismal Studies, Heidelberg University, 69120 Heidelberg, Germany

**Keywords:** Detoxification program, Repression, ROXY-type glutaredoxins, TGA transcription factors

## Abstract

**Background:**

Glutaredoxins (GRXs) are small proteins which bind glutathione to either reduce disulfide bonds or to coordinate iron sulfur clusters. Whereas these well-established functions are associated with ubiquitously occurring GRXs that encode variants of a CPYC or a CGFS motif in the active center, land plants also possess CCxC/S-type GRXs (named ROXYs) for which the biochemical functions are yet unknown. ROXYs physically and genetically interact with bZIP transcription factors of the TGA family. In Arabidopsis, ectopically expressed ROXY19 (originally named GRX480 or GRXC9) negatively regulates expression of jasmonic acid/ethylene-induced defense genes through an unknown mechanism that requires at least one of the redundant transcription factors TGA2, TGA5 or TGA6.

**Results:**

Ectopically expressed ROXY19 interferes with the activation of TGA-dependent detoxification genes. Similar to the *tga2 tga5 tga6* mutant, *35S:ROXY19* plants are more susceptible to the harmful chemical TIBA (2,3,5-triiodobenzoic acid). The repressive function of ROXY19 depends on the integrity of the active site, which can be either CCMC or CPYC but not SSMS. Ectopic expression of the related GRX ROXY18/GRXS13 also led to increased susceptibility to TIBA, indicating potential functional redundancy of members of the *ROXY* gene family. This redundancy might explain why *roxy19* knock-out plants did not show a phenotype with respect to the regulation of the TIBA-induced detoxification program. Complementation of the *tga2 tga5 tga6* mutant with either TGA5 or TGA5_C186S,_ in which the single potential target-site of ROXY19 had been eliminated, did not reveal any evidence for a critical redox modification that might be important for controlling the detoxification program.

**Conclusions:**

ROXY19 and related proteins of the *ROXY* gene family can function as negative regulators of TGA-dependent promoters controlling detoxification genes.

**Electronic supplementary material:**

The online version of this article (doi:10.1186/s12870-016-0886-1) contains supplementary material, which is available to authorized users.

## Background

Plants live in challenging environments in which they have to recognize different stress cues and initiate appropriate responses. The activation of stress-opposing genes is accomplished by the action of transcriptional regulators that alter gene expression patterns to favor the anti-stress program over other metabolic processes.

The three related class II-TGA transcription factors TGA2, TGA5 and TGA6 and their interacting transcriptional co-activator SCARECROW-LIKE (SCL) 14 are required for the activation of genes of the detoxification program after plants encounter potentially harmful chemicals [1–3]. Moreover, these TGA factors require the interacting redox-regulated regulatory protein NPR1 (NON EXPRESSOR OF PR-GENES1) to activate the plant immune response “systemic acquired resistance” [4].

In addition to interacting with transcriptional co-activators, TGA factors directly interact with land-plant specific glutaredoxins (GRXs) [5–7], also known as CC-type [8] or class III GRXs [9]. Class III GRXs differ from the well characterized class I and class II GRXs by their active site, which is CCxC/S rather than CPYC (class I) or CGFS (class II). Like the canonical GRXs, they might function as oxidoreductases or as iron-sulfur cluster (Fe-S) binding proteins [10]. The model plant *Arabidopsis thaliana* encodes 21 class III *GRX* genes, which are named *ROXYs* [11].

Similar to the *ROXY* gene family, the family of TGA factors has expanded during evolution and specific members play distinct roles in development, metabolism and defense. Examples for a functional connection between TGAs and ROXYs have been found for each of these three processes. In development, ROXY1 negatively regulates TGA transcription factor PERIANTHIA (PAN) to control meristematic activity during floral organogenesis [12]. In microsporogenesis, ROXY1 and ROXY2 operate in one pathway with TGA factors TGA9 and TGA10 [13]. In nitrate metabolism, a functional connection between ROXY11 to ROXY15 with TGA1 and TGA4 seems likely [14]. Finally, ten of the 17 tested ROXYs interfere with transcriptional activation of the master regulator of an important anti-microbial defense pathway, *ORA59* [7]. This defense pathway is activated by the stress hormones jasmonic acid (JA) and ethylene (ET) and contributes to fending off necrotrophic pathogens [15, 16]. The *ORA59* promoter contains an essential TGA binding site (TGACGT) which is occupied in vivo by class II-TGA factors [17]. By interfering with *ORA59* expression, ectopically expressed ROXY19 blocks the activation of JA/ET-induced defense processes and confers higher susceptibility to the necrotrophic pathogen *Botrytis cinerea* [7, 18]. Since *ROXY19* is induced at the transcriptional level by the defense hormone salicylic acid (SA), it was suggested to be responsible for the conserved negative effect of SA on the JA/ET pathway [5].

In this study, we addressed the question whether other yet unknown processes are influenced by ROXY19. Due to the potential redundancy of members of the large *ROXY* gene family, we deployed transgenic plants expressing *ROXY19* under the control of the constitutive *Cauliflower Mosaic Virus 35S* (*CaMV 35S*) promoter. We took an unbiased approach and analysed the transcriptomes of unchallenged whole seedlings. Gene Ontology (GO) term and Motif Mapper analysis of the negatively regulated genes revealed that TGA-dependent genes potentially involved in the detoxification of reactive chemicals were repressed by ROXY19. Consistently, *35S:ROXY19* plants are more susceptible to the halogenated electrophilic xenobiotic TIBA.

## Results

### Microarray analysis identified novel genes regulated by ROXY19

**Fig. 1:**
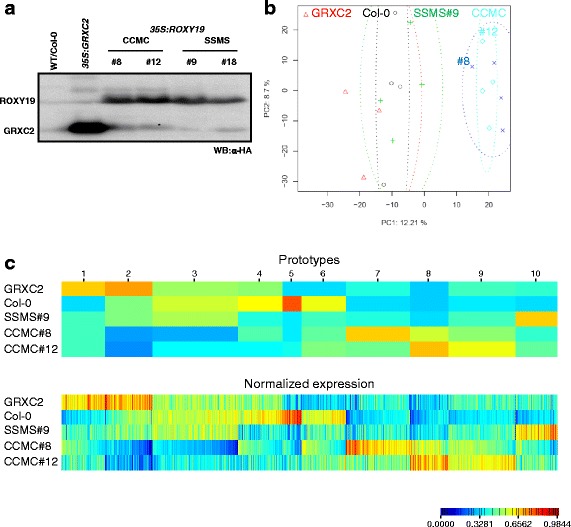
**a** Western blot analysis to detect HA-tagged proteins in plant lines subjected to microarray analyses.*35S:ROXY19*_*SSMS*_ line #18 was only used later for transcript analysis by qRT-PCR. **b** Principal component analysis of the normalized transcriptome data. Symbols: O, ∆, +, X and ◊, represent biological replicates of WT/Col-0, *35S:GRXC2*, *35S:ROXY19*_*SSMS*_ line #9, *35S:ROXY19* l ine #8 and *35S:ROXY19* line #12, respectively. **c** Clustering of 1486 differentially expressed in at least of the four transgenic lines. Genes were clustered into 10 prototypes according to their normalized expression pattern using the MarVis software (*upper panel*). The width of each prototype column is proportional to the number of genes assigned to this prototype. The lower panel shows the normalized expression profiles of the individual transcripts. The program color codes the relative expression of a given prototype (upper panel) or transcript (lower panel) in the five genotypes. Red depicts the highest relative expression, blue the lowest (see color scale)

In order to identify further genes whose expression can be influenced by ROXY19, we performed microarray analysis of two independent transgenic plant lines (#8 and #12) expressing *ROXY19* under the control of the *CaMV 35S* promoter. We chose axenically grown seedlings rather than soil-grown plants for this analysis in order to reduce the variability that comes with fluctuations in the environment. Moreover, roots could be harvested along with the shoot without any stressful treatments during the up-rooting process. We included RNA from transgenic plants expressing a putatively non-functional ROXY19 in which the conserved CCMC motif was mutated to SSMS (ROXY19_SSMS_). In parallel, RNA from a transgenic line expressing class I GRXC2 (formerly called GRX370), which does not interact with TGA factors [5], was analysed. All proteins were N-terminally fused to an HA-tag and expression of similar amounts of protein in each line was confirmed by Western blot analysis (Fig. 1a). Untransformed Col-0 plants and the four transgenic plant lines were grown on vertically oriented agar plates and 2-week-old seedlings (roots and shoots) were harvested for RNA extraction. The experiment was repeated four times with independently grown plant material.

In order to get a first impression of the global structure of the dataset of our microarray investigation, we performed a principal component analysis (PCA) which typically results in clusters of samples with a similar expression pattern (Fig.1b). The samples from Col-0 and transgenic *35S:ROXY19*_*SSMS*_ plants did not show a clear separation indicating that the ROXY19_SSMS_ protein does not strongly alter the transcriptome. In contrast, the clusters representing the two independent *35S:ROXY19* lines overlapped and were separated from the Col-0 and *35S:ROXY19*_*SSMS*_ clusters. These results provide evidence for the requirement of a functional active site during transcriptional regulation by ROXY19. The data set derived from the *35S:GRXC2* plants was also clearly distinct from the data set obtained from the *35S:ROXY19* plants indicating a specific function of ROXY19 as opposed to a potential general function associated with glutaredoxins.

Additional file 1: Table S1 and Additional file 2: Table S2 list those genes that were differentially expressed (fold change (FC) <0.74 or >1.37, *p <* 0.05) in the two *35S:ROXY19* lines but not in lines *35S:GRXC2* or *35S:ROXY19*_*SSMS*_. Col-0 was taken as the reference sample. According to these criteria, 299 and 291 transcripts were significantly more abundant in line #8 and line #12, respectively (Additional file 1: Table S1); 337 and 246 transcripts were less abundant (Additional file 2: Table S2).

To visualize and cluster the relative transcript levels of all those genes that are differentially expressed (fold change (FC) <0.74 or >1.37, *p <* 0.05) in at least one of the four transgenic lines when compared to Col-0, we applied the MarVis software (Fig. 1c; [19]). This program groups genes with similar relative expression levels into prototypes and color-codes the relative expression levels in the five genotypes. For example, Prototype 1 comprises genes that are up-regulated in all transgenic lines. Importantly, prototypes 2 and 3 contain those genes that are less expressed only in the two *35S:ROXY19* lines and not in the two other transgenic lines. Prototype 8 contains 136 genes that are up-regulated only in the two *35S:ROXY19* lines.

In summary, this analysis shows a robust negative effect of ROXY19 on target genes in both transgenic lines, which is consistent with its known repressive function on promoters driven by or containing a functional *as-1* element [5, 7, 17]. Up-regulated genes do not show such a consistent clustering in the two *35S:ROXY19* lines. This might indicate that positive effects on gene expression are more indirect and thus more subject to fluctuations. Therefore, we mainly focused on the down-regulated genes. Since the repressive effect seemed stronger in line #8, the following analysis was done with the 337 transcripts (corresponding to 321 genes) down-regulated in these plants.

### TGA binding sites are enriched in promoters that are repressed by ROXY19

Next, we tested whether binding sites for transcription factors are over- or under-represented in the promoters of differentially expressed genes. To this end, the 1-kb sequences upstream of the predicted transcriptional start sites were scanned using the Motif Mapper *cis* element analysis tool ([20]; Table 1). The program determines the average number of specific binding sites in a given group of genes that is randomly (1000 times) selected from the whole genome. This average frequency is compared with the actual number of binding sites within the group of ROXY19-regulated genes. Strikingly, the TGACG motif, which represents more than a half site of the perfect TGA binding site TGACGTCA [21, 22] and which is recognized by TGA2 [23], is present in 226 promoters belonging to the 337 less abundant transcripts in line #8. This motif is enriched by a factor of 1.58 (Table 1). The TGACGT motif, which covers 6 bps of the palindrome, is present in 140 promoters and enriched by a factor of 2.45. The perfect palindrome TGACGTCA [21] is enriched even 6.19-fold, although only present in 27 of the down-regulated promoters. Table 1 presents all the motifs which we identified as being enriched after applying the following three criteria. First, the motif should be present in at least 10 % of the promoters of the differentially expressed genes; second the motif should be enriched by at least 1.5-fold; third, the *p-*value should be 0. In addition to the TGACG and the TGACGT motifs, variants of the A box (TTACGT and TTACGTA) and the C box (CACGTC) were identified. Binding of TGA factors (TGA1a) to A boxes TACGTA and C boxes (GACGTC) was shown before [24]. Moreover, the A box overlaps with the preferred binding site of ATAF-type NAC factors (TTACGTA [25]). Notably, transcript levels of *ATAF1* and *ANAC032*, which have been shown before to be regulated by the SCL14/TGA complex [1], are reduced in both transgenic lines ectopically expressing ROXY19 (Additional file 2: Table S2). Thus, the over-representation of the A box might reflect that ATAF1/NAC032-dependent promoters are indirectly affected by ROXY19.

**Table 1:** Promoter elements enriched in promoters affected in *35S:ROXY19* plants (line #8)

	35S:ROXY19#8		Average selection		Ratio: total motifs	*p-*value
Motif	Promoter hits	Total motifs	Promoter hits	Total motifs		
TGACG	226	439	173.8	278.0	1.58	0
TG**ACGT**	140	214	69.5	87.0	2.45	0
TT**ACGT**	123	193	95.2	126.8	1.52	0
T**ACGT**C	56	58	24.8	26.7	2.17	0
TT**ACGT**A	49	72	28.2	36.6	1.97	0
TG**ACGT**CA	27	27	4.2	4.4	6.19	0
CATGCAY	84	122	55.6	76.3	1.60	0
TTTATATA	120	175	84.4	114.4	1.53	0
TAAAATAT	106	157	77.9	104.4	1.50	0

The occurrence of enriched motifs was determined in the 1-kb sequences upstream of the 5′-untranslated regions. Upper panel: Analysis of 321 promoters belonging to 337 transcripts that are less abundant in *35S:ROXY19* than in Col-0 plants. Lower panel: Analysis of 292 promoters belonging to the 299 transcripts that are more abundant in *35S:ROXY19* plants than in Col-0 plants. Numbers represent the total number of promoters (promoter hits) that contain the indicated motif or the total amount of motifs within the set of promoters affected by ROXY19 and within randomly chosen sets of promoters from the whole genome. With the exception of the TGACGTCA motif, only elements are shown that occur in at least 10 % of the selected promoters and which are enriched by at least 1.5-fold and *p =* 0. The ACGT core sequence is shown in bold in order to document that variants of this sequence with different flanking bases are enriched

Analysis of the promoters of the up-regulated genes revealed that AT-rich sequences resembling a TATA box are enriched (Table 1). In addition, the sequence CATGCAY, which represents a RY motif that is recognized by B3-domain transcription factors [26] is present with an enhanced frequency in the up-regulated promoters.

### Plants ectopically expressing ROXY19 are more susceptible to xenobiotic stress

**Fig. 2:**
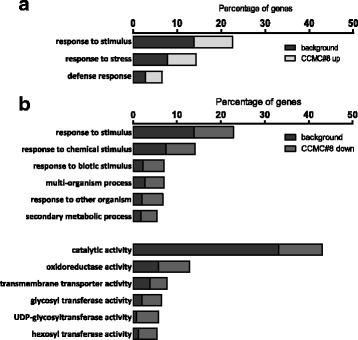
xGene Ontology (GO) over-representation analysis of genes which show enhanced (**a**) or reduced (**b**) expression in *35S:ROXY19* (line #8) plants. Black bars indicate the percentage of genes of each GO term found within the group of all annotated genes of the Arabidopsis genome. Gray bars indicate the percentage of genes of each GO term found within the subgroup of differentially regulated genes. Only GO terms encompassing more than 17 genes (5 %) are shown

In order to identify the function of the target genes of ROXY19, we subjected the differentially expressed genes to Gene Ontology (GO) over-representation analysis (Fig. 2 [27]). Only GO terms encompassing more than 5 % of the genes were considered. The GO-terms “response to stimulus”, “response to stress” and “defense response” were significantly enriched, irrespective of whether the up-regulated or the down-regulated genes were analysed (Fig. 2). In contrast, only members of the group of down-regulated genes were enriched within the GO domain “molecular function”. Here, the GO term “catalytic activity” and its sub-GO terms “oxidoreductase activity” (phase 1 of the detoxification process), “glycosyl transferase activity”, “UDP-glycosyltransferase activity” and “hexosyl transferase activity” (phase 2 of the detoxification process) were enriched. Moreover, the GO term “transmembrane transporter” (phase 3 of the detoxification process) was over-represented. Thus, the GO term enrichment analysis suggests that ectopically expressed ROXY19 suppresses the detoxification response.

The TGA-dependent detoxification pathway is induced by chemicals like the xenobiotic TIBA (2,3,5-trijodobenzoic acid [1]), the allelochemical BOA (benzoxazolin-2(3H)-one [28, 29]), the safeners isoxadifen-ethyl and mefenpyr-diethyl [2] and phytoprostanes [3]. Therefore, we hypothesized that at least a fraction of the ROXY19-repressed genes should be induced by these chemicals. Therefore, we analysed the expression levels of the 337 down-regulated transcripts (corresponding to 321 genes) in mock-treated and TIBA-treated plants. To this aim, we deployed a data set obtained from a previously conducted microarray analysis. Since the microarray was performed with the ATH1 gene chip, we only obtained expression data for 301 genes affected by ROXY19. Indeed, 101 out of these 301 genes were induced by TIBA after 8 h (Additional file 3: Table S3).

**Fig. 3:**
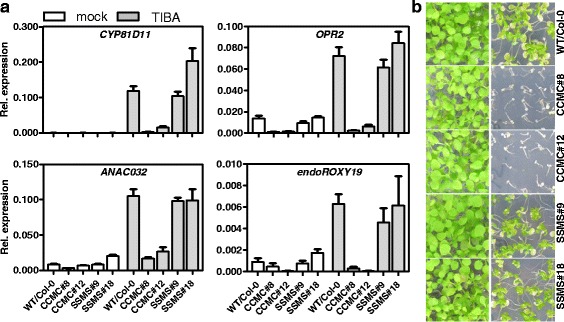
**a** Quantitative RT-PCR analysis of TIBA-inducible genes in independent transgenic lines expressing comparable amounts of either ROXY19 or ROXY19_SSMS_ as shown by Western blot analysis (see Fig. 1). Four-week-old soil-grown plants of the indicated genotypes were either sprayed with 0.1 mM TIBA/0.05 % DMSO or 0.05 % DMSO (mock). Leaves were harvested for RNA isolation after 10 h of treatment. Relative transcript levels were determined using *UBQ5 as a* reference gene. The mean values (+/−SE) obtained from four to five individually harvested plants are shown. **b **Growth phenotypes of the plant lines used in (**a**) on MS plates containing 1 % sucrose and 50 μM TIBA/0.025 % DMSO (right panel) or 0.025 % DMSO (*left panel*). Photographs were taken two weeks after germination

Next we tested the repressive capacity of ROXY19 on selected target genes using quantitative reverse transcription (qRT)-PCR analysis. We chose *CYP81D11*, *OPR (OPDA REDUCTASE) 2* and *ANAC032* for this analysis, since these genes were (1) strongly repressed in *35S:ROXY19* lines, (2) induced by TIBA (see Additional file 3: Table S3), and (3) less induced in the TIBA-treated *tga2 tga5 tga6* mutant ([1] and Additional file 4: Figure S1). Moreover, all three genes are related to the detoxification program: *CYP81D11* [30] might play a role in detoxification of the oxylipin 9-HOT (9-hydroxy-10,12,15-octadecatrienoic acid), *OPR2* [31] in detoxification of the explosive TNT (2,4,6-trinitrotoluene) and network analysis has identified *ANAC032* as a central activator of detoxification genes [32]. All three genes were only barely inducible in soil-grown *35S:ROXY19* plants whereas ROXY19_SSMS_ allowed wild-type-like transcript levels upon TIBA treatment (Fig. 3). This documents that ROXY19 exerts its negative effect not only in axenically grown plantlets. Using primers directed against the endogenous *ROXY19* transcripts, we reproduced previous findings that ectopically expressed ROXY19 represses its own gene [33]. Moreover, the experiment confirmed publicly available expression data (Genevestigator, [34]) and our microarray analysis that *ROXY19* is induced by TIBA.

Given the overlap of ROXY19-repressed genes with TIBA-induced genes and the results of the GO term analysis, we tested whether plants ectopically expressing ROXY19 would be more susceptible to TIBA. To this aim, seedlings were grown on 50 μM TIBA (Fig. 3b). As observed before for the *tga2 tga5 tga6* triple mutant [1], *35S:ROXY19* seedlings were more impaired in their growth and showed stronger signs of bleaching than wild-type plants and the two *35S:ROXY19*_*SSMS*_ lines. This effect was also detected with soil-grown plants, albeit the phenotypic differences were less evident (Additional file 5: Figure S2). Therefore, all subsequent TIBA-susceptibility assays were performed with plants grown on plates.

### The CCMC active site can be changed to CPYC in ROXY19

**Fig. 4:**
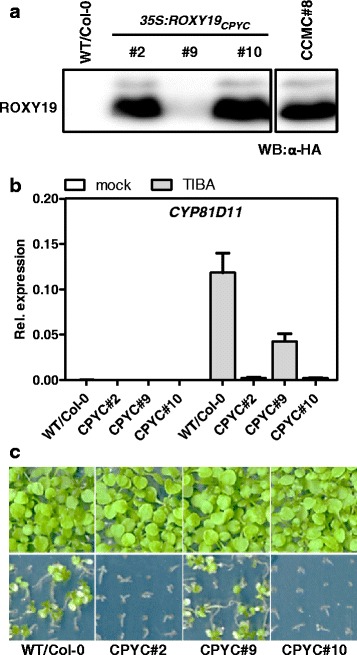
**a** Western blot analysis to detect HA-tagged ROXY19_CPYC_. For comparison, a protein extract from *35S:ROXY19* line #8 was loaded on the same blot (lanes with non-relevant samples were excised). **b** Quantitative RT-PCR analysis of *CYP81D11* in independent transgenic lines expressing either ROXY19 or ROXY19_CPYC_. Four-week-old soil-grown plants of the indicated genotypes were either sprayed with 0.1 mM TIBA/0.05 % DMSO or 0.05 % DMSO (mock). Leaves were harvested for RNA isolation after 10 h of treatment. Relative transcript levels were determined using *UBQ5 as a* reference gene. The mean values (+/−SE) obtained from four to five individually harvested plants are shown. **c** Growth phenotypes of the plant lines analysed in (**a**) and (**b**) on MS plates containing 1 % sucrose and 50 μM TIBA/0.025 % DMSO (lower panels) or 0.025 % DMSO (*upper panels*). Photographs were taken two weeks after germination

Next, we mutated the conserved CCMC motif of ROXY19 into a CPYC motif that is found in class I GRXs. Two transgenic lines expressing the ROXY19_CPYC_ protein to similar levels as those found in the previously characterized transgenic line #8 and one line expressing lower amounts were selected for further analysis (Fig. 4a). The two highly expressing lines showed reduced expression of *CYP81D11* (Fig. 4b) after TIBA treatment which correlated with higher susceptibility to TIBA (Fig. 4c). In contrast, line #9 with lower ROXY19_CPYC_ protein levels showed only slightly reduced *CYP81D11* expression and wild-type-like sensitivity of the growth phenotype on TIBA-containing MS plates (Fig. 4c). It is concluded that the repression mechanism requires an active site, but not precisely the characteristic CCMC motif.

### The detoxification pathway is not hyper-induced in the *roxy19* mutant

**Fig. 5:**
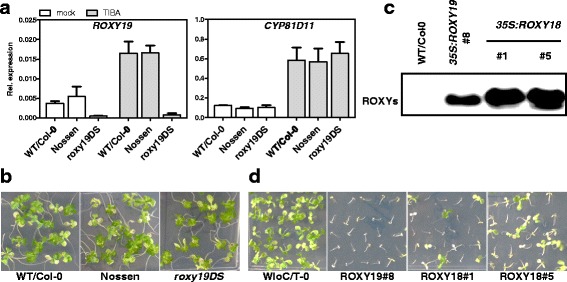
**a** Quantitative RT-PCR analysis of *ROXY19* and *CYP81D11* in *roxy19DS* mutant plants. Two-week-old MS plates-grown plants of the indicated genotypes were transferred onto MS plates supplemented with 0.1 mM TIBA/0.05 % DMSO or 0.05 % DMSO (mock). Plants were harvested for RNA isolation after 48 h of treatment. Relative transcript levels were determined using *UBQ5 as a* reference gene. The mean values (+/−SE) obtained from three to four biological replicates (one replicate corresponds to a pool of 40 to 50 plantlets) are shown. **b** Growth phenotypes of the plant lines analysed in (**a**) on MS plates containing 1 % sucrose and 50 μM TIBA/0.025 % DMSO. Photographs were taken two weeks after germination. **c** Western blot analysis to detect HA-tagged ROXY18. For comparison, a protein extract from *35S:ROXY19* line #8 was loaded on the same blot. **d** Growth phenotypes of the plant lines analysed in (**c**) on MS plates containing 1 % sucrose and 50 μM TIBA/0.025 % DMSO. Photographs were taken two weeks after germination

Microarray analysis of mock- and TIBA-treated plants had revealed that only the expression of *ROXY19* (Additional file 6: Figure S3) is induced by TIBA. Moreover, our studies have shown that ROXY19 can negatively affect the expression of TIBA-induced detoxification genes. These results suggest that endogenous ROXY19 might counteract the activation process through a negative feedback loop. Therefore, we tested whether target genes of ROXY19 would be expressed to higher levels in a *roxy19* knock-out mutant and whether the mutant plants would be more resistant to TIBA. Since no T-DNA insertion line is available in the Col-0 ecotype, we deployed the transposon-tagged line *roxy19DS* (ecotype Nossen) in which the Ds element is inserted 45 bps upstream of the ROXY19 start codon. This insertion interferes with *ROXY19* mRNA accumulation as demonstrated by qRT-PCR analysis of RNA collected from wild-type and mutant seedlings (Fig. 5a). Still, TIBA-induced *CYP81D11* transcript levels were indistinguishable between wild-type and the mutant (Fig. 5a). Moreover, the sensitivity towards TIBA remained unchanged (Fig. 5b).

In order to address the question whether other ROXYs might act redundantly with ROXY19, we tested transgenic plants expressing ROXY18 (Fig. 5c), which is the closest relative to ROXY19 [12]. Ectopic expression of ROXY18 to the same levels than ROXY19 resulted in similar increased susceptibility to TIBA (Fig. 5d).

### The single cysteine in class II TGA factors does not affect its biological activity

**Fig. 6:**
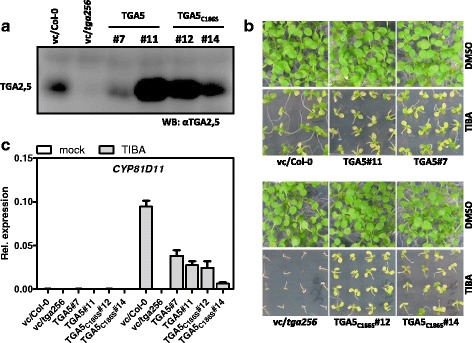
**a** Immunological detection of ectopically expressed TGA5 or TGA5_C186S_ in the *tga2 tga5 tga6* (*tga256*) background with a TGA-specific antiserum. vc, empty vector control. **b** Growth phenotypes of the plant lines analysed in (**a**) on MS plates containing 1 % sucrose and supplemented 50 μM TIBA TIBA/0.025 % DMSO (lower panels) or 0.025 % DMSO (upper panels). Photographs were taken two weeks after germination. **c** Quantitative RT-PCR analysis of *CYP81D11* in independent transgenic lines expressing either TGA5 or TGA5_C186S_. Four-week-old soil-grown plants of the indicated genotypes were either sprayed with 0.1 mM TIBA/0.05 % DMSO or 0.05 % DMSO (mock). Leaves were harvested for RNA isolation after 10 h of treatment. Relative transcript levels were determined using *UBQ5 as a* reference gene. The mean values (+/−SE) obtained from four to five individually harvested plants are shown.vc, vector control plants

Since TGA factors interact with ROXY-type GRXs it may be speculated that ROXYs regulate TGA factor activity through modulating their redox state. Class II TGA factors encode a single conserved cysteine within the domain C-terminal to the bZIP domain. In order to analyse whether the redox state of this cysteine might be of functional relevance, we complemented the *tga2 tga5 tga6* triple mutant with either wild-type TGA5 or a TGA5 mutant protein that encodes a serine rather than a cysteine residue at amino acid position 186 (TGA5_C186S_). Expression of the transgene was verified by Western blot analysis with an antiserum that recognizes TGA2, TGA5 and TGA6. With the exception of *35S:TGA5* line #7, transgenic lines accumulated higher levels of TGA protein than Col-0 (Fig. 6a). In spite of these different degrees of gene expression, all four lines complemented the increased TIBA susceptibility of the *tga2 tga5 tga6* triple mutant with similar efficiencies (Fig. 6b). However, the complementing TGA5 constructs did not fully restore TIBA-induced *CYP81D11* expression. This phenomenon was independent of whether the TGA factor was mutated or not. Expression was also not altered in mock-treated samples (Fig. 6c).

## Discussion

ROXY19 belongs to a 21-membered gene family of proteins which are classified as glutaredoxins. ROXY19 was found in a modified yeast screen using bZIP transcription factor TGA2 bound to the TGACGT motif as a bait [5]. Ectopically expressed ROXY19 represses the JA/ET pathway by interfering with the expression of the transcriptional master regulator *ORA59* [7]. Since TGA factors bind in vivo to the *ORA59* promoter [17], it was speculated that ROXY19 is recruited to the promoter through its interaction with TGA factors. Indeed, direct recruitment of ROXY19 to its own TGA-regulated promoter was shown by chromatin immunoprecipitation analysis [33].

In this study, we asked the question whether ROXY19 can target further promoters. To this end, transgenic plants ectopically expressing HA-tagged ROXY19 were subjected to microarray analysis. Consistent with the idea that ROXY19 negatively regulates class II TGA factors, genes containing TGACGT motifs in their promoter regions were repressed. GO term analysis revealed that ROXY19-down-regulated genes might serve to detoxify reactive chemicals. This assumption is derived from the observation that genes representing catalytic activities typical for the three phases of the detoxification process [35] are over-represented. First, oxidoreductases might reduce carbonyl to hydroxyl groups in the first phase, which serves to activate compounds for conjugation processes in phase 2. Indeed, (UDP) glucosyl-transferases were over-represented. In phase 3, these compounds are transported to the vacuole or the apoplast. Consistently, transmembrane transporters were also enriched in the set of genes that are less well expressed in *35S:ROXY19* plants. The impaired growth of *35S:ROXY19* plants on TIBA-containing MS plates/1 % sucrose can be explained by the reduced expression of these genes. However, it is very likely that further target genes can be identified by analyzing the transcriptomes of soil-grown plants either treated with a combination of those signals that induce *ROXY19* expression like treatment with SA, JA, SA/JA, or sodium chloride (roots) or infection with pathogens.

Based on the phenotype of the *35S:ROXY19* lines, it was expected that the detoxification pathway might be hyper-activated in *roxy19DS* plants. However, expression of target genes of ROXY19 was not altered. Since the mutant was generated in the Nossen background and since the transposon insertion is upstream of the ATG start codon, results may not yet be conclusive. Therefore, studies with a *roxy19* allele in the Columbia background, which have become feasible with the advent of the CRISPR-Cas-mediated genome editing technology, have to be awaited.

Still, potential redundancy of other ROXYs has to be taken into account especially in consideration of the increased TIBA-susceptibility of *35S:ROXY18* plants. Likewise, expression of a number of ROXYs under the *ROXY1* promoter can restore the wild-type flower phenotype in the *roxy1* background [12]. Moreover, at least 10 ROXYs can repress the *ORA59* promoter in transient assays [7]. In both experimental systems, all functional ROXYs encode a conserved ALWL motif at their very C-terminal end. Therefore, the hypothesis was put forward that ROXYs might principally operate through the same mechanism and that their specific expression pattern determines when and where they become functional. This concept seems likely for reproductive tissues, where the expression of developmental genes in specific cell types follows a fine-tuned protocol. However, in differentiated leaves, several *ROXYs* are constitutively expressed (e.g. the potentially redundant ROXY10 and ROXY18 proteins (Additional file 6: Figure S3). Thus, TIBA-induced expression of *ROXY19* should only slightly increase the amount of potentially redundant ROXYs.

**Fig. 7:**
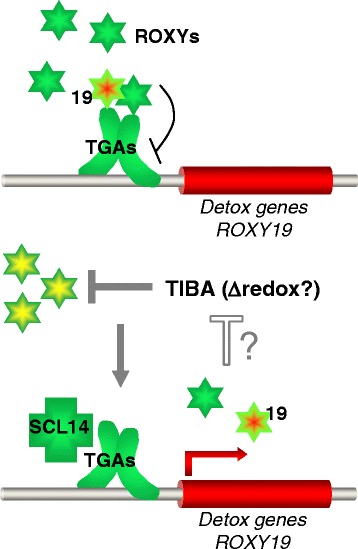
Model of the role of ROXYs as repressors of the detoxification pathway. We postulate that redundant ROXYs bind to class II TGA factors at the promoters of detoxification genes repressing their activation by SCL14 [1] or other transcriptional co-activators. Upon TIBA treatment, the repressive capacity of ROXYs is abolished allowing activation of gene expression. This inactivation might be incomplete in plants expressing high amounts of ROXYs. Since control of gene expression is often subject to autoregulatory mechanisms, newly synthesized ROXYs including TIBA-induced ROXY19 might negatively regulate the inactivation mechanism in order to turn off the response. Ectopic expression of repressive ROXYs might interfere with this intricate mechanisms leading to permanent repression

We therefore do not claim a specific role for ROXY19 but rather hypothesize that basal levels of redundant ROXYs might repress the detoxification pathway in the absence of a toxic compound (Fig. 7), similar to the repressive activities of redundant JAsmonate ZIM-domain (JAZ) proteins in the absence of JA [36, 37]. Xenobiotic chemicals would inactivate ROXYs through post-transcriptional (redox?) modifications. To reconcile this hypothesis with the constitutive repressive capacity of ectopically expressed ROXY18 and ROXY19, we have to postulate that the inactivation might involve a mechanism that is not effective if the pool of ROXYs is too large. ROXY19 inhibits own expression as well as the expression of *ROXY18* (ROXY18 is the most stringently down-regulated gene in both *35S:ROXY19* plant lines; see Additional file 2: Table S2). This suggests that the expression levels of these proteins have to be tightly controlled since abnormally high expression levels might lead to permanent repression. Given that regulatory circuits are often interwoven, it might even be that ROXYs can interfere with the inactivation mechanism. These assumptions would explain the strong and permanent repressive capacity of ectopically expressed ROXY19. This hypothesis might be challenged by higher order mutants or transgenic lines expressing a dominant negative ROXY protein.

Furthermore, we observed that ROXY19 interferes with TGA-regulated processes if the active site is CCMC or CPYC. However, mutating the site into an SSMS sequence abrogated its function. Thus, either oxidoreductase activity or assembly of an iron sulfur (Fe-S) cluster might be important for repression. Until now, biochemical analyses are only available for a His-tagged CC-type GRX from poplar in which the hydrophobic ALWL motif at the C terminus had been replaced by the C-terminal amino acids of poplar class I GRXC4 [10]. Absorption peaks of the recombinant protein measured at 322 and 415 nm indicated the association with a 2Fe-2S cluster. However, the protein had poor enzymatic activity with the artificial substrate 2-hydroxyethyldisulfide. Whether TGA factors are redox-modified by ROXYs has remained an open question. ROXY1 requires the first cysteine of the CCMC active site to complement the *roxy1* phenotype [38]. In addition, the TGA transcription factor PAN cannot complement the *pan* flower phenotype if a critical cysteine residue is mutated into serine [12]. This might indicate that the oxidized version of PAN is the active protein and that ROXY1 interferes with its function by catalyzing the reduction. In the case of class II TGA factors, we found that the pseudo-reduced protein TGA5_C186S_ was still functional: the target genes *CYP81D11* was repressed in the absence of TIBA and activated to wild-type-like levels in the presence of TIBA. Therefore, we consider it unlikely that the redox state of TGA factors is crucial with respect to the regulation of the detoxification pathway.

Since the CPYC sequence does not compromise the ability of ROXY19 to repress TGA-regulated genes, the importance of the CCMC sequence has remained elusive. One hypothesis is that the CCMC motif is important for the relief of the repressive activities.

## Conclusion

Glutaredoxins are of major importance for sensing the redox state of cells and relaying the signal to a wide variety of metabolic and regulatory processes. In the evolution of higher plants, particularly the CC-type GRXs have undergone a major expansion resulting in large gene families such as the 21-membered ROXY family in Arabidopsis. While the biochemical and molecular functions of CC-type GRXs are still largely unknown, a functional link with TGA transcription factors in the regulation of reproductive organ development, nitrate metabolism and defense pathways has been established. We have identified the detoxification pathway as another process ROXY proteins may be involved in. Both, ROXY18 and ROXY19 have the potential to negatively regulate TGA-dependent expression of genes characteristic for the three phases of the detoxification process. These findings further emphasize the close functional relation between ROXY proteins and TGA transcription factors. Furthermore, we have shown that the cysteines in the active site of ROXY19 are required for this function. This suggests that in this context ROXY19 may function as an oxidoreductase, which, however, very likely does not act on class II TGA factors. Identification of potential targets of ROXY-mediated redox modification will be a matter of future research.

## Methods

### Plant material and growth conditions

*Arabidopsis thaliana* accession Columbia (Col-0) was used as a wild-type and transgenic plants were in the Col-0 background. The *tga6-1 tga2-1 tga5-1* triple mutant [4] was obtained from Y. Zhang (University of British Columbia, Vancouver, Canada). AGI codes of the genes analyzed or discussed are found in Additional file 8: Table S5. The Ds transposon insertion line (RATM16-0018-1) in the *ROXY19* (At1g28480) locus was obtained from RIKEN, Japan. Homozygous insertion lines were identified with primers LP2GxROXY19, DS3-2a and RPGxROXY19 (Additional file 7: Table S4). If not indicated otherwise, axenically grown plants were cultivated at a 14-h-light/10-h-dark regime at 22–24/18–20 °C with 80 to 100 μmol photons m^−2^ s^−1^. For microarray analysis, seedlings were grown on vertically oriented agar plates containing Murashige and Skoog (MS) medium. For growth assays in the presence of TIBA, seedlings were grown on MS plates containing 1 % sucrose and supplemented with 50 μM TIBA in 0.025 % dimethyl sulfoxide (DMSO) or 0.025 % DMSO. For assessment of the TIBA-susceptibility phenotype, sucrose was included since the growth of plants expressing functional ROXYs and the *tga2 tga5 tga6* mutant was variable even without TIBA. 16 ~ 25 seeds of each genotype were placed onto a sub-square in square Petri dishes allowing the comparison of nine genotypes on one plate. The arrangement of different lines on the different sub-squares was randomized in different biological replicates to exclude edge effects. For the assessment of TIBA-induced gene expression (Fig. 5), about 50 seeds were sown on vertically positioned agar plates containing MS medium. After 12 days, plants were transferred to MS-plates containing 0.05 % DMSO or 0.1 mM TIBA/0.05 % DMSO and incubated for 48 h. Alternatively (Figs 3, 4 and 6), plants were grown for four weeks on steamed soil (Archut, Fruhstorfer Erde, T25, Str1fein) in growth chambers (21/19 °C, 16-h-light/8-h-dark cycle) with light intensity at 80 to 100 μmol photons m^−2^ s^−1^ and 60 % humidity. Plants were sprayed with either 0.1 mM TIBA/0.05 % DMSO or with 0.05 % DMSO and leaves were harvested after 10 h.

### Construction of recombinant plasmids and stable plant transformation

The Gateway technology (Invitrogen, Karlsruhe, Germany) was used to generate plasmids for the ectopic expression of proteins in stable transformants. pB2-HA-GW7 originates from the binary vector pB2GW7.0 (http://www.psb.ugent.be/) containing the expression cassette of pE-35S-HA-GW7 [39]. The generation of plasmid pB2-HA-ROXY19, pB2-HA-ROXY19_CPYC_, pB2-HA-ROXY18, pB2-HA-GRXC2/GRX370, and pB2-TGA5 was already described [7, 40]. Generation of the ROXY19_SSMS_ derivative was achieved by PCR using primer pairs p1/p3 and p2/p4 and pDONR201/ROXY19 as a template [5] resulting in two fragments which served as templates for overlapping PCR with primers p1 and p2. The PCR product was recombined into pB2-HA-GW7. Likewise, the C186S mutation in TGA5 was achieved by generating two PCR products on pDONR201/TGA5 using primer pairs (p1/p5 and p2/p6) and subsequent amplification by overlapping PCR (primers p1 and p2) and recombination into pB2GW7. The sequences of the primers are listed in 6file 5: Table S4. Sequencing confirmed that the mutations had been introduced as planned. For generation of transgenic plants, binary plasmids were electroporated into *Agrobacterium tumefaciens* strain GV3101 (pMP90). The resulting agrobacteria were used to transform Col-0 plants using the floral dipping method [41]. Seeds obtained from F2 plants were used for the analysis. With the exception of *35S:ROXY18* plants, all analysed plants were homozygous.

### Microarray analyses

RNA was extracted by the Trizol method and samples were hybridized with Arabidopsis GeneChipGene 1.0 ST Arrays (Affimetrix) according to [42]. Robust Multi-array Average (RMA)-normalized data, fold change values, and *p-*values derived from moderated t-statistics were obtained from the Affymetrix CEL files using the Robin 1.1.2 software [43]. For *cis* element enrichment analyses, the algorithm Cluster Analysis Real Randomization incorporated into Motif Mapper Version 5.2.4.01 [20] was deployed to define significant distribution alterations compared to 1000 randomly composed, equally sized, reference promoter datasets. For more details, see [17]. The AgriGO program was used for the functional classification of differentially expressed genes [27].

### Quantitative Reverse Transcription (qRT)-PCR and Western blot analysis

RNA extraction and qRT-PCR analyses were performed as described [1]. Calculations were done according to the 2^–Δ*C*T^ method [44] using the *UBQ5* (*At3g62250*) gene as a reference [45]. Primers serving to amplify and quantify transcript levels are indicated in Additional file 7: Table S4. Expression of HA-tagged proteins in stably transformed plants was confirmed by Western blot analysis. Protein extracts were prepared in 450 μl extraction buffer (4 M urea, 16.6 % glycerol, 5 % SDS, 0.5 % β-mercaptoethanol) per 150 mg plant material. Protein concentrations were determined using the Pierce 660 nm assay kit (Thermo Fisher Scientific Inc., Rockford, IL USA). 15 μg were loaded on a 12 % SDS gel. Proteins were detected using either the HA-antibody (Santa Cruz Biotechnology, Inc., Santa Cruz, USA) or the αTGA2/5 antiserum [5] and the Amersham ECL™ Advance Western Blotting Detection Kit (GE Healthcare Europe GmbH, Munich, Germany).

## Additional files:

Additional file 1: Table S1. Microarray data of genes with higher expression levels (FC > 1.3, *p <* 0.05) in *35S:ROXY19* plants as compared to control plants. The table contains the gene identity (AGI), description, the mean expression values of four independent biological replicates of the genotypes Col-0, *35S:GRXC2*, *35S:ROXY19*_*SSMS*_, *35S:ROXY19#8* and *35S:ROXY19#12*, and the ratios (FC, fold changes, log2) of the transcript levels in the transgenic lines with respect to Col-0 and the corresponding *p-*values. (XLSX 163 kb) Additional file 2: Table S2. Microarray data of genes with lower expression levels (FC < 0.74, *p <* 0.05) in *35S:ROXY19* plants as compared to control plants. The table contains the gene identity (AGI), description, the mean expression values of four independent biological replicates of the genotypes Col-0, *35S:GRXC2*, *35S:ROXY19*_*SSMS*_, *35S:ROXY19#8* and *35S:ROXY19#12*, and the ratios (FC, fold changes, log2) of the transcript levels in the transgenic lines with respect to Col-0 and the corresponding *p-*values. (XLSX 305 kb) Additional file 3: Table S3. Microarray data of genes with lower expression levels (<0.74, *p <* 0.05) in *35S:ROXY19* plants as compared to control plants and their response to TIBA in the Col-0 background. The table contains the gene identity (AGI), description, the mean expression values of four independent biological replicates of the genotypes Col-0 and *35S:ROXY19#8*, the ratios (FC, fold changes, log2) of the transcript levels in the transgenic line with respect to Col-0 and the corresponding *p-*values, the ratios between Col-0 treated with 0.1 % DMSO and Col-0 treated with 0.1 mM TIBA/0.1 % DMSO and the corresponding *p-*values. Since the microarray analysis of the TIBA-treated plants was performed with the Affimetrix ATH1 gene chip, the list contains 301 and not 321 genes. Genes that are not induced by TIBA are shown in light grey. FC, fold changes. For TIBA induction, plants were grown for six to seven weeks on steamed soil (Archut, Fruhstorfer Erde, T25, Str1fein) in growth chambers with light intensity at 37 to 45 μmol photons m^−2^ s^−1^ at 22 °C and 60 % humidity. Eight plants were sprayed with either 0.1 mM TIBA/0.1 % DMSO or with 0.1 % DMSO and leaves were harvested after eight h. The experiment was repeated three times. (XLSX 205 kb) Additional file 4: Figure S1. Quantitative RT-PCR analysis of *OPR2* and *ANAC032* transcript levels in TIBA-treated wild-type and *tga2 tga5 tga6* mutant plants. Four-week-old soil-grown plants of the indicated genotypes were either sprayed with 0.1 mM TIBA/0.05 % DMSO or 0.05 % DMSO (mock). Leaves were harvested for RNA isolation after 10 h of treatment. Relative transcript levels were determined using *UBQ5 as a* reference gene. The mean values (+/−SE) obtained from three to five individually harvested plants are shown. (PPTX 107 kb) Additional file 5: Figure S2. Growth phenotypes of plant lines Col-0, 35S:ROXY19, 35S:ROXY19_SSMS_ and *tga2 tga5 tga6* after TIBA treatment. Plants were grown for three weeks on steamed soil (Archut, Fruhstorfer Erde, T25, Str1fein) in growth chambers (21/19 °C, 16-h-light/8-h-dark cycle) with light intensity at 80 to 100 μmol photons m^−2^ s^−1^ and 60 % humidity. Plants were sprayed with 0.2 mM TIBA/0.1 % DMSO or with 0.1 % DMSO for 4 times with a 24- h interval each. Photographs were taken one day after the last treatment. (PPTX 17227 kb) Additional file 6: Figure S3. Relative expression values from different *ROXYs* as determined by microarray analysis of RNA from leaves from mock- and TIBA-treated plants. For plant treatments, see legend to Additional file 3, Table S3. The relative fluorescence intensities (transformed to a linear scale) representing potentially redundant *ROXYs* encoding an ALWL motif at the C terminus were plotted. (PPTX 67 kb) Additional file 7: Table S4. List of primer sequences (DOCX 11 kb) Additional file 8: Table S5. List of AGI codes of the genes analysed or discussed in this study. (DOCX 10 kb)

## Abbreviations

9-HOT: 9-hydroxy-10,12,15-octadecatrienoic acid
BOA: Benzoxazolin-2(3H)-one
CaMV 35S: Cauliflower Mosaic Virus 35S
Col-0: *Arabidopsis thaliana* accession Columbia
DMSO: Dimethyl sulfoxide
ET: Ethylene
FC: Fold change
Fe-S: Iron-sulfur cluster
GO: Gene Ontology
GRX: Glutaredoxins
JA: Jasmonic acid
JAZ: Jasmonate ZIM-domain
MS: Murashige and Skoog
NPR1: NON EXPRESSOR OF PR-GENES1
OPR: OPDA REDUCTASE
PAN: PERIANTHIA
PCA: Principal component analysis
qRT-PCR: Quantitative Reverse Transcription PCR
RMA: Robust Multi-array Average
SA: Salicylic acid
SCL: SCARECROW-LIKE
TIBA: 2,3,5-triiodobenzoic acid
TNT: 2,4,6-trinitrotoluene
