# Supplementary figures and images for: Ectopically expressed glutaredoxin ROXY19 negatively regulates the detoxification pathway in Arabidopsis thaliana
Source: BMC Plant Biol. 2016 Sep 13;16(1):200. doi: 10.1186/s12870-016-0886-1 (PMC5022239; doi:10.1186/s12870-016-0886-1)

## Slide 1
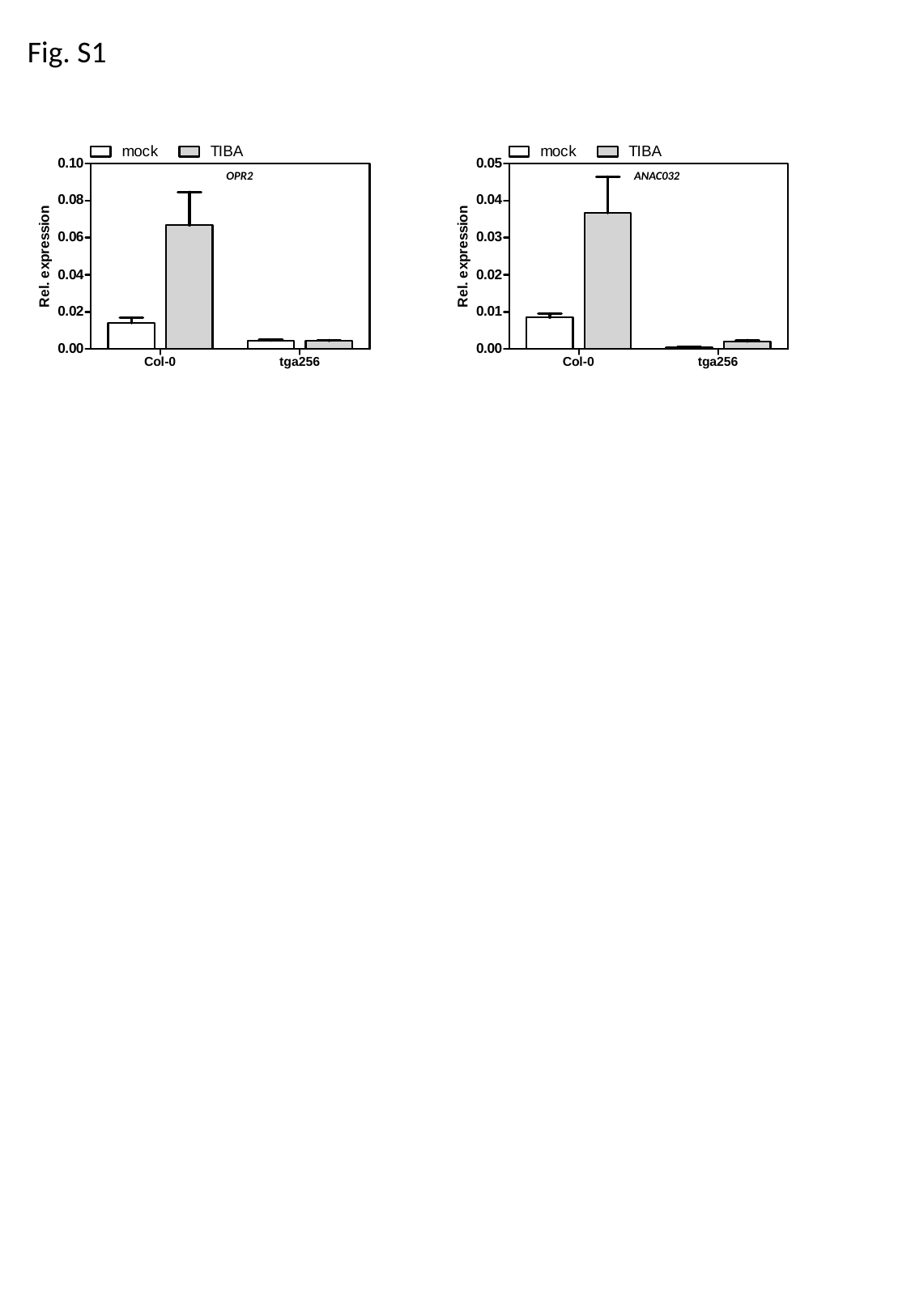

Fig. S1
OPR2
ANAC032

Supplement: Additional file 4: Figure S1. — Quantitative RT-PCR analysis of OPR2 and ANAC032 transcript levels in TIBA-treated wild-type and tga2 tga5 tga6 mutant plants. Four-week-old soil-grown plants of the indicated genotypes were either sprayed with 0.1 mM TIBA/0.05 % DMSO or 0.05 % DMSO (mock). Leaves were harvested for RNA isolation after 10 h of treatment. Relative transcript levels were determined using UBQ5 as a reference gene. The mean values (+/−SE) obtained from three to five individually harvested plants are shown. (PPTX 107 kb) [file 12870_2016_886_MOESM4_ESM.pptx]

## Slide 1
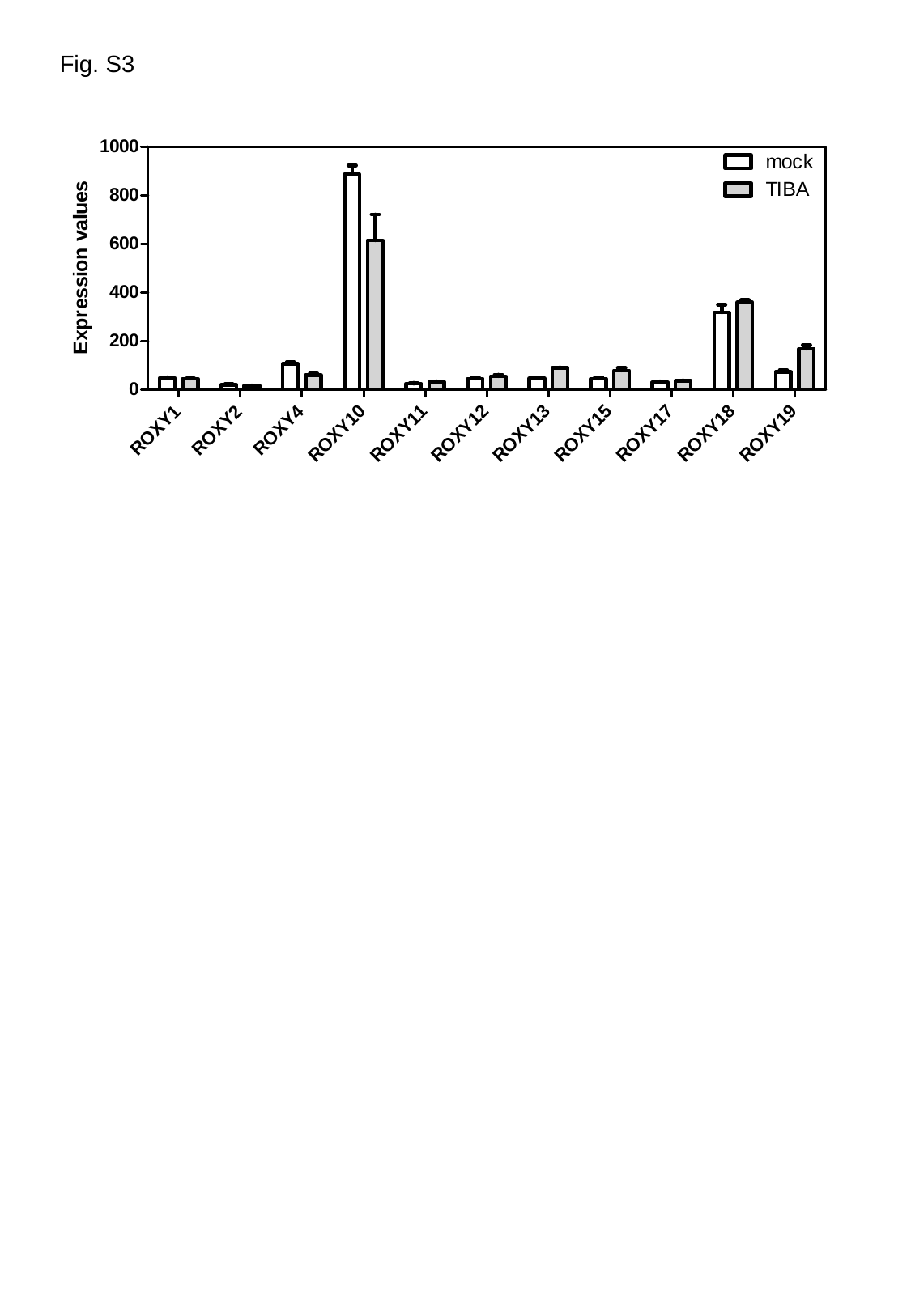

Fig. S3

Supplement: Additional file 6: Figure S3. — Relative expression values from different ROXYs as determined by microarray analysis of RNA from leaves from mock- and TIBA-treated plants. For plant treatments, see legend to Additional file 3, Table S3. The relative fluorescence intensities (transformed to a linear scale) representing potentially redundant ROXYs encoding an ALWL motif at the C terminus were plotted. (PPTX 67 kb) [file 12870_2016_886_MOESM6_ESM.pptx]
