# Supplementary material for: Ectopically expressed glutaredoxin ROXY19 negatively regulates the detoxification pathway in Arabidopsis thaliana
Source: BMC Plant Biol. 2016 Sep 13;16(1):200. doi: 10.1186/s12870-016-0886-1 (PMC5022239; doi:10.1186/s12870-016-0886-1)
Supplement: Additional file 5: Figure S2. — Growth phenotypes of plant lines Col-0, 35S:ROXY19, 35S:ROXY19SSMS and tga2 tga5 tga6 after TIBA treatment. Plants were grown for three weeks on steamed soil (Archut, Fruhstorfer Erde, T25, Str1fein) in growth chambers (21/19 °C, 16-h-light/8-h-dark cycle) with light intensity at 80 to 100 μmol photons m−2 s−1 and 60 % humidity. Plants were sprayed with 0.2 mM TIBA/0.1 % DMSO or with 0.1 % DMSO for 4 times with a 24- h interval each. Photographs were taken one day after the last treatment. (PPTX 17227 kb) [file 12870_2016_886_MOESM5_ESM.pptx]

## Slide 1
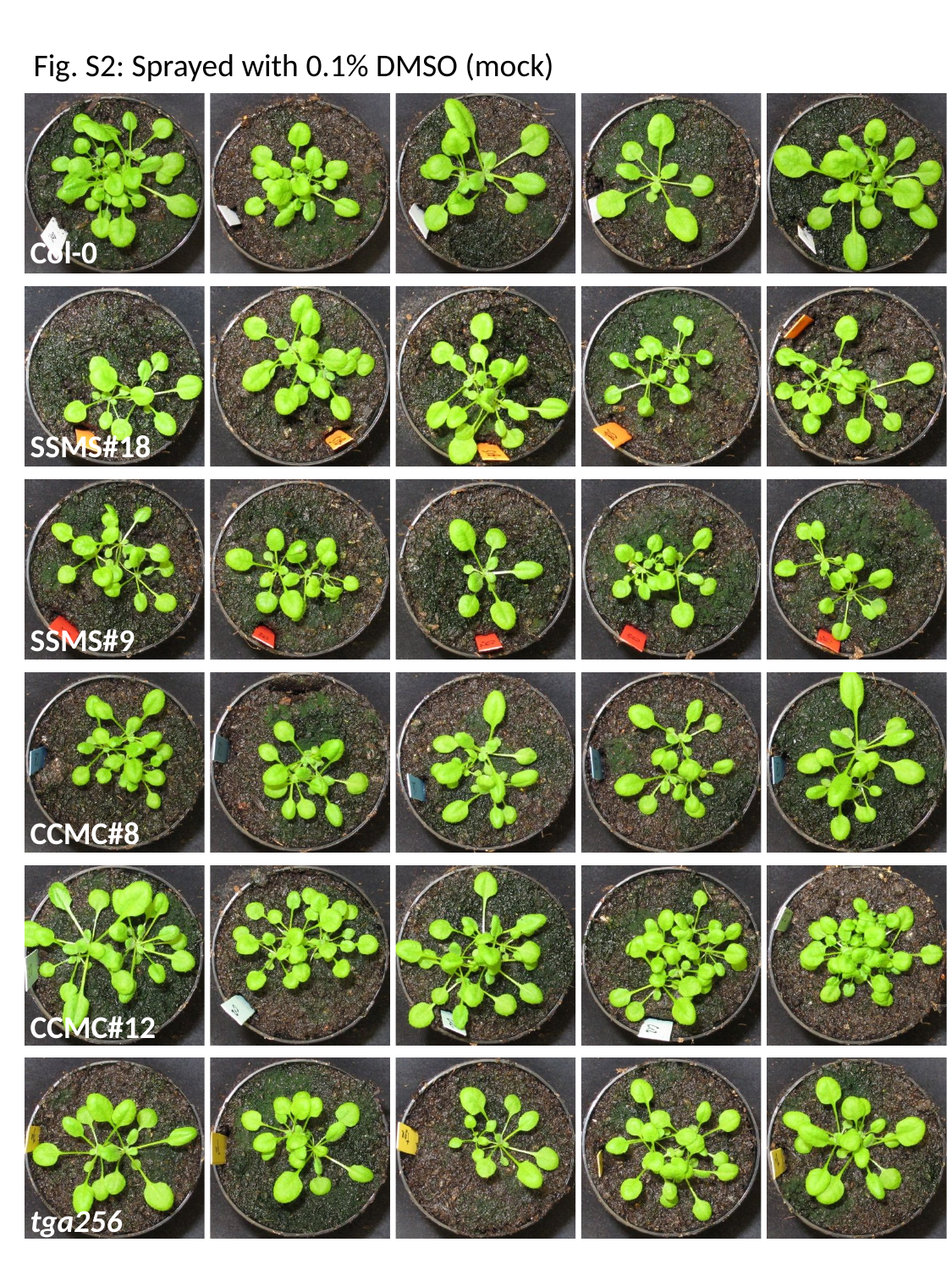

Fig. S2: Sprayed with 0.1% DMSO (mock)
Col-0
SSMS#18
SSMS#9
CCMC#8
CCMC#12
tga256

## Slide 2
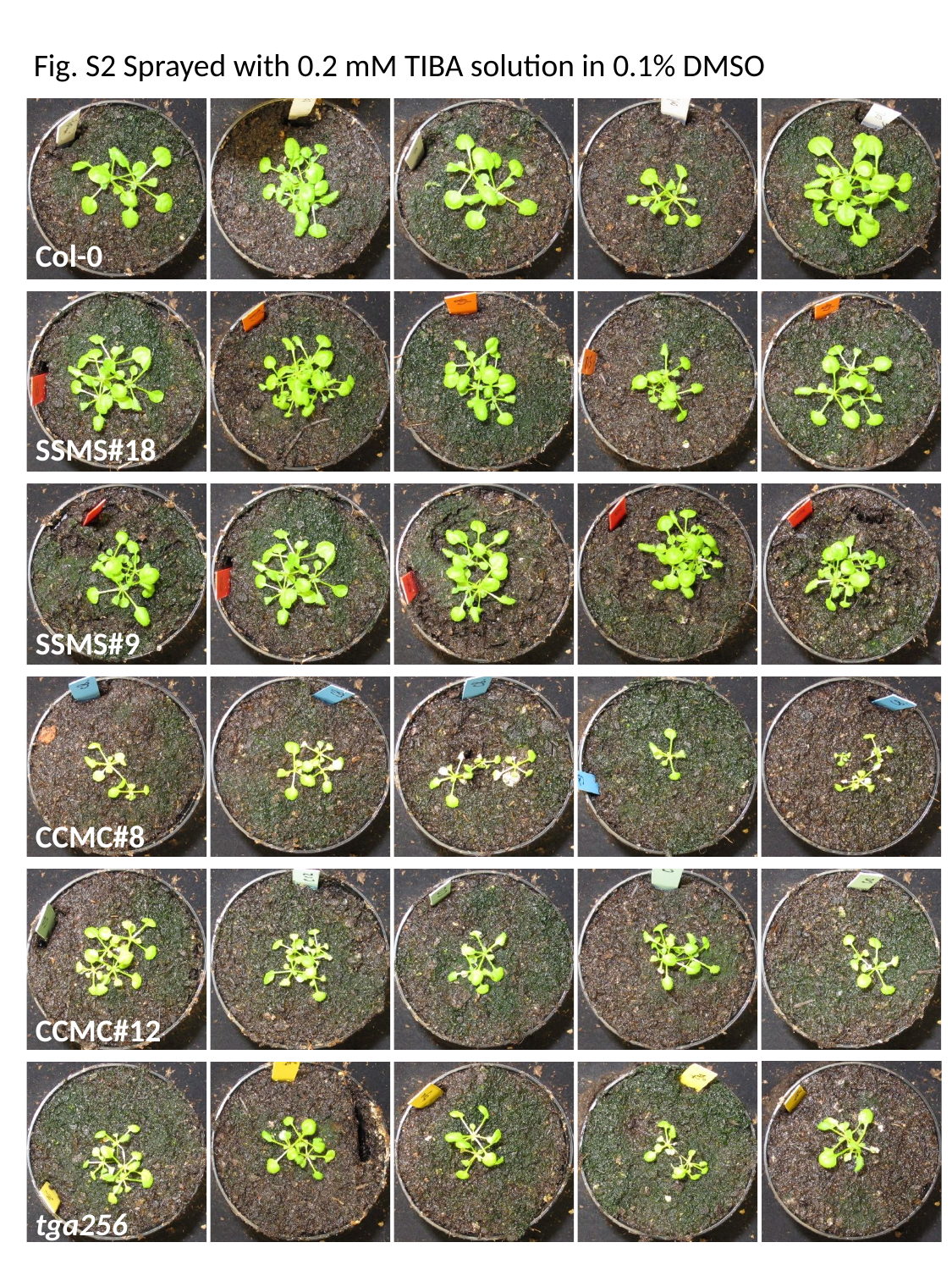

Fig. S2 Sprayed with 0.2 mM TIBA solution in 0.1% DMSO
Col-0
SSMS#18
SSMS#9
CCMC#8
CCMC#12
tga256
