# Supplementary material for: Ectopically expressed glutaredoxin ROXY19 negatively regulates the detoxification pathway in Arabidopsis thaliana
Source: BMC Plant Biol. 2016 Sep 13;16(1):200. doi: 10.1186/s12870-016-0886-1 (PMC5022239; doi:10.1186/s12870-016-0886-1)
Supplement: Additional file 7: Table S4. — List of primer sequences (DOCX 11 kb) [file 12870_2016_886_MOESM7_ESM.docx]

Table S4: List of primers

| primers | Sequences (5’-3) |
| --- | --- |
| Genotyping of transposon insertion line RATM16-0018-1 | |
| LP2GxROXY19 | GATTTTTGATCACTAAGAATATAATGTC |
| Ds3-2a | CCGGATCGTATCGGTTTTCG |
| RPGxROXY19 | CCTCATCAATCTCAAGGACCG |
| Plasmid cloning | |
| p1 | TCGCGTTAACGCTAGCATGGATCTC |
| p2 | GTAACATCAGAGATTTTGAGACAC |
| p3 | CGGAGAGGATCTTCCATGTCTCATGTGG |
| p4 | CCACATGAGACATGGAAGATCCTCTCCG |
| p5 | CCAGCTGAGAGATCTTTCTTGTGG |
| p6 | CCACAAGAAAGATCTCTCAGCTG |
| Quantitative RT PCR analysis | |
| CYP81D11 | QT00781662 (Qiagen) |
| OPR2 | QT00894768 (Qiagen) |
| ANAC032 | QT00743561(Qiagen) |
| ROXY19 | QT00869715 (Qiagen) |
| endogenous ROXY19 | TTGGAGGGTTAGATAGGGTTATGG CGTAAACAACAATTACCAATCAAGATTC |
| UBQ5 | GACGCTTCATCTCGTCC  GTAAACGTAGGTGAGTCCA |
