# Supplementary material for: Ectopically expressed glutaredoxin ROXY19 negatively regulates the detoxification pathway in Arabidopsis thaliana
Source: BMC Plant Biol. 2016 Sep 13;16(1):200. doi: 10.1186/s12870-016-0886-1 (PMC5022239; doi:10.1186/s12870-016-0886-1)
Supplement: Additional file 8: Table S5. — List of AGI codes of the genes analysed or discussed in this study. (DOCX 10 kb) [file 12870_2016_886_MOESM8_ESM.docx]

**List of AGI codes**

ROXY1/GRXC7 AT3g02000

ROXY2/GRXC8 AT5g14070

ROXY4/GRXC11 AT3g62950

ROXY10/GRXS2 AT5g18600

ROXY11/GRXS3 AT4g15700

ROXY12/GRXS5 AT4g15690

ROXY13/GRXS4 AT4g15680

RORY15/GRXS8 AT4g15660

ROXY17/GRXS6 AT3g62930

ROXY18/GRXS13 AT1g03850

ROXY19/GRX480/GRXC9 AT1g28480

TGA5 AT5g06960

GRXC2/GRX370 AT5g40370

CYP81D11 AT3g28740

OPR2 AT1g76690

ANAC032 AT1g77450
